# Parental Emotion Regulation, Psychological Distress, and Family Involvement in Education Among Chilean Caregivers: An Observed-Variable Analysis

**DOI:** 10.3390/bs16101834

**Published:** 2026-10-08

**Authors:** Gerardo Fuentes-Vilugrón, Carlos Arriagada-Hernández, Felipe Caamaño-Navarrete, Francisco Correa-Araneda, Paulo Etchegaray-Pezo

**Affiliations:** 1 Pedagogía en Educación Física, Facultad de Educación, Universidad Católica de Temuco, Temuco 4780000, Chile; gerardo.fuentes@uct.cl; 2 Educación Parvularia, Plan de Prosecución de Estudios, Facultad de Educación, Universidad San Sebastián, Valdivia 5110466, Chile; 3 Pedagogía en Educación Física, Facultad de Educación, Universidad Autónoma de Chile, Temuco 4780000, Chile; carlos.arriagada@uautonoma.cl (C.A.-H.); felipe.caamano@uautonoma.cl (F.C.-N.); 4 Escuela de Terapia Ocupacional, Facultad de Salud, Universidad Santo Tomás, Temuco 4780000, Chile; 5 Centro de Gestión y Tecnologías del Agua, Facultad de Ingeniería y Ciencias, Universidad de la Frontera, Temuco 4811230, Chile; 6 Pedagogía en Inglés and Departamento de Didáctica y Práctica, Facultad de Educación, Universidad Católica de Temuco, Temuco 4780000, Chile; petchegaray@uct.cl

**Keywords:** family involvement in education, emotional regulation, mental health, gender, mediation, moderation, family involvement, psychological distress

## Abstract

This study examined the association between parental emotion regulation (ER) and family involvement in education (FIE), including the potential mediating role of psychological distress, operationalized through depressive, anxiety, and stress symptomatology (DASS), and the moderating role of caregiver gender, while accounting for sociodemographic variables. A cross-sectional study was conducted with a sample of *n* = 219 Chilean parents/caregivers. The Emotion Regulation Evaluation Scale (RE-MESACTS), the Depression, Anxiety and Stress Scale (DASS-21), and the Family Involvement in Education Scale (EPFE) were administered. A sequential observed-variable analytical strategy combining regression, mediation with non-parametric bootstrap (5000 resamples), moderation, covariate-adjusted models, and complementary sensitivity analyses was employed. ER was positively associated with FIE after accounting for DASS (β = 0.230, *p* = 0.004) and negatively associated with DASS (β = −0.410, *p* < 0.001). However, DASS was not significantly associated with FIE (β = −0.065, *p* = 0.365), and the indirect effect of ER on FIE through DASS was not statistically significant (*B* = 0.026, *p* = 0.374). A statistically significant ER × Gender interaction was observed (*B* = 0.739, *p* < 0.001). Simple-slope analyses showed a positive association among female caregivers (*B* = 0.345, *p* < 0.001) and a negative association among male caregivers (*B* = −0.394, *p* = 0.021). An exploratory complementary multigroup analysis showed that the ER → FIE path differed between gender groups, χ^2^(1) = 7.90, *p* = 0.005. Most sociodemographic covariates were not significantly associated with FIE; however, caregivers of students attending private fee-paying schools reported significantly higher FIE than those associated with public schools ( = 3.162, *p* = 0.045). Overall, ER was positively associated with educational involvement, whereas DASS did not statistically mediate the ER–FIE association. The observed ER × Gender interaction suggests a potential gender-dependent pattern; however, because the male subgroup was small (*n* = 38), the gender-specific estimates should be regarded as preliminary and require replication in larger and more gender-balanced samples. The findings highlight the relevance of parental emotion regulation for family involvement and provide a basis for further investigation of psychological and sociodemographic factors associated with educational involvement.

## 1. Introduction

Family involvement in education is one of the factors most consistently associated with the academic, socioemotional, and behavioral success of children and adolescents (Jeynes, 2017; Castro et al., 2015). Research conducted in diverse cultural contexts demonstrates that when families actively engage in the educational process—whether through home support, communication with the school, or participation in school activities—students show better academic outcomes, greater intrinsic motivation, improved psychosocial adjustment, and a lower risk of school dropout (Pomerantz et al., 2007; Hill & Tyson, 2009; Pérez Murga, 2024; Roldan-Quijije, 2025). However, this involvement does not occur in a vacuum, as it is associated with the psychological and emotional characteristics of primary caregivers, particularly in contexts of high parental demand and social vulnerability (Iwanski et al., 2025).

In Latin America, family involvement in education should be understood within educational systems characterized by persistent socioeconomic inequalities and heterogeneous family–school relationships. Evidence from the region indicates that parental involvement may take multiple forms, including educational support at home, communication with teachers, participation in school activities, and involvement in school decision-making processes. Importantly, opportunities for such involvement are not equally distributed. Socioeconomic conditions, caregivers’ time availability, parental educational expectations and self-efficacy, access to information, and the ways in which schools communicate with and invite families to participate may influence both the intensity and form of parental involvement (Gubbins et al., 2024). Consequently, lower levels of visible school-based participation should not necessarily be interpreted as lower parental commitment to children’s education.

This issue is particularly relevant in Chile, where family involvement constitutes both an educational-policy priority and an equity-related concern. Chilean research has shown that socioeconomic conditions can shape parental expectations and opportunities for participation, particularly among families associated with socioeconomically disadvantaged and public-school contexts (Sánchez Oñate et al., 2016). Likewise, parental educational expectations, perceived self-efficacy, and the quality of information provided by schools have been identified as relevant factors associated with home- and school-based involvement (Gubbins & Otero, 2020). Socioeconomic differences have also been reported in caregivers’ opportunities and willingness to engage in more active forms of school participation (Santander Ramírez et al., 2022). At the policy level, the Chilean educational framework explicitly recognizes family–school–community collaboration as an important component of students’ integral development and educational trajectories (Murua Arroyo et al., 2017). Therefore, family involvement in the Chilean context cannot be understood exclusively as an individual parental disposition; rather, it emerges from the interaction between caregivers’ personal and psychological resources and the socioeconomic and institutional conditions that facilitate or constrain participation.

Parental emotion regulation (PER) emerges as a fundamental construct for understanding variations in Family involvement in education (Edvoll et al., 2023; Mullins & Panlilio, 2023). From the perspective of Gross’s (2015) process model, emotion regulation (ER) refers to the processes by which individuals influence which emotions they experience, when they experience them, and how they express them. In the parental context, these skills may help caregivers manage the stress associated with parenting, maintain positive interactions with their children and school professionals, and persevere in educational support activities even when faced with challenges (Meyer et al., 2014; Pulido et al., 2020; Fuentes-Vilugrón et al., 2022). Parents with greater emotion regulation skills tend to show greater consistency in parenting practices, greater sensitivity to their children’s educational needs, and greater capacity to establish effective collaborations with the school (Bridgett et al., 2015; Zimmer-Gembeck et al., 2021). Conversely, difficulties in emotion regulation are associated with more reactive parenting practices, lower persistence in school support activities, and a higher probability of conflict in family-school interactions (Crandall et al., 2015; De Raeymaecker & Dhar, 2022).

Importantly, the proposed association between parental emotion regulation and family involvement does not imply that caregivers with better emotion regulation will necessarily become more involved in their children’s education. Rather, ER is conceptualized here as one psychological resource that may facilitate caregivers’ ability to initiate and sustain educational involvement when such activities involve frustration, interpersonal conflict, competing demands, or other emotionally challenging situations. This interpretation is consistent with Belsky’s (1984) process model of parenting, which proposes that parental functioning is jointly shaped by parents’ psychological resources, child characteristics, and contextual sources of stress and support. It is also consistent with Hoover-Dempsey and Sandler’s (1997) model of parental involvement, according to which parents’ involvement in their children’s education is shaped by parental role construction, perceived self-efficacy, and invitations or opportunities for participation provided by children and schools. Within this framework, emotion regulation does not replace parental self-efficacy, parenting stress, parent–child relationship quality, or socioeconomic resources; rather, it represents a theoretically distinct psychological resource that may facilitate caregivers’ capacity to translate motivations and opportunities for involvement into sustained educational behaviors.

However, the relationship between parental emotion regulation and educational involvement is complex and may involve both direct and indirect pathways. Conservation of resources theory (Hobfoll, 2001) suggests that psychological distress may reflect the depletion or strain of psychological resources needed to sustain social and educational involvement behaviors. Within the present study, psychological distress is operationalized through the combined depressive, anxiety, and stress symptomatology assessed by the DASS-21. Within this framework, depressive, anxiety, and stress symptomatology could represent one plausible mediating mechanism, as parental emotional difficulties consume psychological resources necessary for maintaining active and sustained educational involvement (Silk et al., 2011). Empirical evidence supports the plausibility of this pathway, showing that parents with depressive or anxious symptoms exhibit less energy to participate in school activities (Mendoza-Burgos & Restrepo-Echavarría, 2022), greater social withdrawal, and more negative perceptions of their ability to influence their children’s education (Goodman et al., 2011; Sweeney & MacBeth, 2016). Thus, it is relevant to examine whether emotion regulation is associated with family involvement not only directly, but also indirectly through psychological distress, as indexed by depressive, anxiety and stress symptomatology.

To integrate these perspectives into a coherent theoretical model, the present study combines three complementary frameworks. First, from Gross’s (2015) process model, parental emotion regulation is conceptualized as a metacognitive competence that allows individuals to modify emotional trajectories in contexts of educational demand. Accordingly, ER is not proposed as a sufficient or exclusive determinant of family involvement, but as one psychological resource that may facilitate the maintenance of involvement behaviors under emotionally demanding conditions. Second, conservation of resources theory (Hobfoll, 2001) suggests that psychological distress, operationalized in this study as the overall depressive, anxiety, and stress symptomatology assessed by the DASS-21, may represent one potential intervening mechanism between personal competencies and involvement behaviors. Higher scores therefore represent greater psychological distress rather than lower psychological well-being. In this sense, DASS symptomatology is examined as one theoretically plausible intervening mechanism rather than as the only possible explanation of the ER–FIE association. Third, from the gender socialization perspective (Chaplin & Aldao, 2013), differential social expectations could moderate how emotion regulation translates into observable behaviors of educational involvement.

In addition to these psychological mechanisms, the relationship between emotion regulation and educational involvement is likely conditioned by contextual and sociodemographic factors (Riquelme et al., 2019; Fuentes-Vilugrón et al., 2023). Particularly, caregiver gender may moderate the association between emotion regulation and educational involvement, given that social expectations, parental roles, and emotional experiences differ systematically between men and women in most societies (Chaplin & Aldao, 2013). The hypothesis that gender moderates the relationship between emotion regulation and Family involvement in education is grounded in three lines of evidence: (a) differential emotional socialization that promotes greater expression and interpersonal regulation of emotions in women (Chaplin & Aldao, 2013); (b) findings that women tend to use more regulation strategies centered on social support (Tamres et al., 2002), which could translate more directly into school involvement behaviors (communication with teachers, participation in meetings); and (c) evidence from Latin American contexts showing gender differences in the expression of family involvement (Oyarzún-Farías et al., 2024). Previous studies suggest that women tend to show greater emotional expression (Gordillo-León et al., 2021) and social support-seeking in contexts of parental stress, while men might use more problem-solving-based regulation strategies (Tamres et al., 2002; Shangguan et al., 2022). These differences could translate into distinct patterns of association between emotion regulation and educational involvement according to caregiver gender.

Despite these theoretical and empirical advances, important gaps persist in the literature. First, most studies have examined the constructs of emotion regulation, mental health, and family involvement in isolation, without integrating them into comprehensive models that allow for the simultaneous evaluation of mediation and moderation relationships. Second, although the literature on family involvement in Latin America has increased, evidence examining the psychological resources associated with caregivers’ educational involvement remains limited. This gap is particularly relevant in Chile, where family participation occurs within a highly differentiated educational system and may be shaped by both socioeconomic and institutional conditions. Consequently, examining psychological correlates of family involvement while simultaneously considering sociodemographic characteristics and school type may provide a more contextually grounded understanding of family–school relationships. Third, relatively few studies have examined these relationships through an integrated analytical strategy combining regression-based mediation, non-parametric bootstrap estimation, interaction-based moderation, covariate-adjusted models, and complementary observed-variable path analyses. Such an approach allows direct, indirect, and conditional associations to be examined within a common analytical framework (Preacher & Hayes, 2008). Fourth, possible suppression effects have not been explored, where the inclusion of a mediating variable could increase, rather than decrease, the relationship between predictor and criterion variables, a phenomenon with important theoretical and practical implications (MacKinnon et al., 2000). The exploratory suppression analysis is justified from the methodological framework of MacKinnon et al. (2000), who point out that a suppressor effect occurs when the inclusion of a third variable increases the magnitude of the coefficient between predictor and criterion, revealing a relationship that had remained ‘hidden’ due to irrelevant variance.

In response to these limitations, the present study aims to examine the association between parental emotion regulation and family involvement in children’s education. Specifically, it seeks to: (a) evaluate the mediating role of depressive, anxiety and stress symptomatology in the relationship between emotion regulation (ER) and family involvement in education (FIE); (b) examine the moderating role of gender in the direct ER-FIE relationship; and (c) examine the associations of relevant sociodemographic characteristics with FIE while including these characteristics as control variables. Additionally, as a complementary exploratory analysis, the possible presence of suppression effects when including DASS in the predictor model of FIE will be examined, although no specific hypothesis is formulated given the exploratory nature of this analysis.

Previous research supports gender-related differences in emotional socialization, emotional expression, and emotion-regulation strategies, as well as differences in patterns of family involvement. However, the available evidence does not establish a consistent direction regarding how caregiver gender may condition the relationship between emotion regulation and family involvement in education. Therefore, a non-directional moderation effect is proposed.

The hypotheses proposed in this research are:

**H1.**  *Psychological distress, operationalized as DASS symptomatology, will partially mediate the relationship between ER and FIE, such that higher levels of emotion regulation will be associated with lower psychological distress and, consequently, with greater Family involvement in education.*

**H2.**  *Caregiver gender will moderate the relationship between ER and FIE, such that the strength of this association will differ between male and female caregivers.*

This study contributes theoretically by integrating within a single analytical framework the constructs of emotion regulation, parental mental health (depression, anxiety, and stress), and family educational involvement within the Latin American context, proposing and evaluating simultaneous mediation and moderation relationships. By examining these relationships in Chile and incorporating sociodemographic characteristics and school type into the analytical strategy, the study also contributes context-specific evidence regarding how caregivers’ psychological resources relate to family educational involvement within a socially and institutionally differentiated educational system. The proposed relationships were examined using observed-variable analyses within a correlational framework, with the specific analytical procedures described in detail in the Statistical Analysis section. Practically, the study may provide preliminary evidence to inform future research examining whether parental emotional competencies constitute modifiable factors relevant to family educational involvement. However, given the cross-sectional design, the present study does not test whether strengthening emotion regulation produces changes in family involvement or psychological distress.

## 2. Materials and Methods

A non-experimental, cross-sectional, correlational study was conducted. A quantitative approach was employed. The proposed relationships were examined using regression-based analyses and observed-variable path models within a correlational framework. Mediation and moderation analyses were conducted using observed total scores rather than latent variables; therefore, no measurement model or latent-variable structural equation model was estimated.

### 2.1. Sample

The sample consisted of *n* = 219 participants (parents or primary caregivers of students) (Table 1). Non-probabilistic convenience sampling was used. Participants were recruited using a snowball sampling technique between July 2025 and March 2026. Initial participants were invited to share information about the study with other potentially eligible caregivers within their social and community networks. Data collection was conducted in Chile, primarily through an online questionnaire. All questionnaire items were configured as mandatory; therefore, submitted questionnaires could not contain item-level missing responses. Consequently, no missing-data imputation was required for the variables included in the analyses. Inclusion criteria were: (a) being a father, mother, or legal guardian of at least one elementary or secondary education student, (b) residing in the same city as the student, and (c) providing informed consent. Participation was voluntary, and all participants received information about the study before providing informed consent. No family contributed more than one caregiver to the analytical sample; therefore, the 219 participants represented 219 independent family units, supporting the assumption of independence of observations at the family level.

Because the final sample size was not determined through an a priori power analysis, a sensitivity power analysis was conducted to characterize the minimum effect size detectable with the available sample. Using G*Power 3.1 (Faul et al., 2009), sensitivity was evaluated for the primary moderation model, in which the ER × Gender interaction constituted the focal predictor. With *n* = 219, α = 0.05, statistical power of 0.80, one tested predictor, and three total predictors (ER, caregiver gender, and ER × Gender), the minimum detectable incremental effect size was f^2^ = 0.036. Thus, the total sample provided sensitivity to detect a relatively small incremental interaction effect under standard multiple-regression assumptions. However, this calculation refers to the overall interaction test and does not imply equivalent precision for the gender-specific slopes. In particular, because the male subgroup comprised only *n* = 38 caregivers, the male-specific conditional estimate may be less precise and stable and is therefore interpreted as preliminary.

### 2.2. Instruments

Psychometric instruments with previous evidence of validity and reliability in Chilean and/or Latin American Spanish-speaking populations were used for measurement. All instruments employed Likert-type scales, whose psychometric properties have been supported in previous research. The selection of instruments was based on their theoretical adequacy for evaluating the constructions in the model and on their reliability and validity indices reported in the specialized literature. All instruments were administered in Spanish. Because validated Spanish-language versions were already available, no translation procedure was conducted specifically for the present study. Previous validation evidence and the psychometric performance observed in the current sample are described below. The specific instruments used in this study are described below.

(a) Emotion Regulation Evaluation Scale (RE-MESACTS): Designed to assess emotion regulation ability. The instrument consists of 15 items with a 4-point Likert-type response format (1 = never to 4 = always), distributed across four factorial dimensions: (a) Positive Emotion Expression (EP); (b) Negative Emotion Self-Regulation (AN); (c) Self-Regulation Strategies (EA); (d) Emotion Identification (IE). The RE-MESACTS was developed in Spanish and psychometrically validated across six Latin American countries, including Chile. Its validation involved multiple samples of adolescents and young people aged 13–20 years and supported a 15-item, four-factor structure, as well as stable internal consistency, replicability, and functioning across cultural contexts (MESACTS, 2020). Although previous validation evidence includes Chilean participants, the original validation samples did not specifically comprise adult caregivers. The total score is obtained by summing the responses, with a theoretical range of 15 to 60 points. According to the authors, scores are interpreted as: 15–38 (low emotion regulation), 39–41 (vulnerable emotion regulation), and 42–60 (normal or high emotion regulation) (MESACTS, 2020). In the present sample, the total RE-MESACTS score demonstrated acceptable internal consistency (Cronbach’s α = 0.792; McDonald’s ω = 0.794). Reliability coefficients for the subscales ranged from α = 0.738 to 0.877 and from ω = 0.742 to 0.880, indicating acceptable to good internal consistency across dimensions (Table 2).
(b) Family Involvement in Education Scale (EPFE): The instrument developed and validated by Sánchez Oñate and Reyes (2023) consists of 20 items with a 4-point Likert-type response format ranging from 1 = never to 4 = always. The items are organized into three dimensions: Home Support (10 items), Democratic Participation (6 items), and School-Family Communication (4 items). Scores are obtained by summing up the corresponding items, with higher scores indicating greater family involvement in education. The total score therefore ranges theoretically from 20 to 80 points. The original validation study was conducted with 308 parents and caregivers from a public primary school in the Biobío Region, Chile. Content validity was assessed by 13 academic experts, and construct validity analyses supported a three-factor structure explaining 42.91% of the variance. The final 20-item scale showed adequate internal consistency (Cronbach’s α = 0.862), with reliability coefficients of α = 0.769 for Home Support, α = 0.743 for Democratic Participation, and α = 0.755 for School-Family Communication. A split-half reliability coefficient of 0.848 was also reported. Because the instrument was originally developed and validated in Spanish in Chile, no translation or linguistic adaptation was required for the present study (Sánchez Oñate & Reyes, 2023) (Table 2).
(c) Depression Anxiety Stress Scale (DASS-21): DASS-21 assesses the absence, presence, and intensity of depression, anxiety, and stress symptoms (Cirilo-Acero et al., 2025). Each subscale consists of seven items, scored from 0 to 3, determining a maximum score of 21 per dimension. Its application focuses on a recent time period. It has three subscales: Depression (items: 3, 5, 10, 13, 16, 17, and 21), Anxiety (items: 2, 4, 7, 9, 15, 19, and 20), and Stress (items: 1, 6, 8, 11, 12, 14, and 18). To evaluate each subscale separately, the scores of the corresponding items for each must be summed. A general indicator of emotional symptoms can be obtained by summing the scores of all items (Daza et al., 2002). A Chilean Spanish version of the DASS-21 has previously demonstrated adequate psychometric properties. In a validation study involving 484 Chilean university students, Antúnez and Vinet (2012) reported evidence supporting a three-factor structure, as well as concurrent and divergent validity and adequate internal consistency. The reported Cronbach’s alpha coefficients were 0.85 for Depression, 0.73 for Anxiety, 0.83 for Stress, and 0.91 for the total scale (Antúnez & Vinet, 2012). For the purposes of the present study, the DASS-21 total score was used as an observed indicator of psychological distress, reflecting the overall burden of depressive, anxiety, and stress symptomatology. Higher total scores indicate greater psychological distress. Accordingly, throughout the manuscript, the term “psychological distress” refers specifically to this operationalization and is not used as an inverse measure of positive psychological well-being. In the present sample, the DASS-21 total score demonstrated excellent internal consistency (Cronbach’s α = 0.914; McDonald’s ω = 0.915). Reliability coefficients for the three subscales were also high, ranging from α = 0.889 to 0.907 and from ω = 0.894 to 0.908 (Table 2).

### 2.3. Analysis Procedure

Analyses were performed using R version 4.3.0 (R Foundation for Statistical Computing, Vienna, Austria), RStudio version 2025.09.2+418 (Posit Software, PBC, Boston, MA, USA), and jamovi version 2.6.45.0. The psych package (version 2.6.5) and ggplot2 package (version 4.0.3) in R were used for descriptive and psychometric analyses and graphical visualization, whereas jamovi was used for the revised regression, mediation, covariate, moderation, multigroup path, and alternative-model analyses.

The analytical strategy followed a theory-driven sequential approach using observed variables within a correlational framework. The term “sequential” refers to the order in which the hypothesized and complementary analyses were conducted and is not intended to imply a novel methodological approach. First, the main association between ER and FIE was examined. Second, the hypothesized indirect pathway through psychological distress, operationalized using the DASS-21 total score, was tested (H1). Third, sociodemographic variables were incorporated as covariates in a separate adjusted model to assess the robustness of the proposed association. Fourth, the moderating role of caregiver gender in the ER–FIE relationship was examined (H2). Complementary multigroup, alternative directional, and suppression analyses were subsequently conducted to assess the robustness and specificity of the main findings. Throughout the manuscript, unstandardized regression and path coefficients are denoted by B, whereas standardized coefficients are denoted by β. Standard errors (SE) and 95% confidence intervals (CI) are reported for unstandardized coefficients where applicable. Indirect, direct, and total effects in the mediation analysis, as well as interaction coefficients and conditional simple slopes in the moderation analysis, are reported in unstandardized form and are therefore denoted by B. The lowercase letters a, b, and c′ used in the mediation model refer exclusively to conventional path labels and should not be confused with coefficient notation. The analyses were conducted in the following sequence:

(a) Descriptive statistics: Means, standard deviations, skewness, and kurtosis were calculated for all continuous variables. Absolute skewness values > 2 and absolute kurtosis values > 7 were considered indicators of severe deviation from normality (West et al., 1995).
(b) Main Association between ER and FIE: Before testing the hypothesized mediation and moderation effects, a linear regression model was estimated with the total FIE score as the outcome and the total ER score as the predictor. This analysis was conducted to establish the main ER-FIE association before testing the proposed mediating and moderating mechanisms.
(c) Mediation Analysis: To evaluate H1, the proposed indirect pathway through psychological distress was tested using the total scores of ER, DASS, and FIE as observed variables. ER was specified as the predictor (X), DASS as the mediator (M), and FIE as the outcome (Y), thereby estimating the ER → DASS pathway (a), the DASS → FIE pathway controlling for ER (b), the direct ER → FIE effect (c′), and the indirect effect (a × b). Non-parametric bootstrap estimation with 5000 resamples was used to obtain 95% confidence intervals for the indirect effect. An indirect effect was considered statistically significant when its bootstrap confidence interval did not include zero (Preacher & Hayes, 2008).
(d) Model with Sociodemographic Covariates: Following evaluation of the main and mediated relationships, age, educational level, number of children, and school type were entered as covariates predicting FIE to assess whether the ER–FIE association remained robust after accounting for these characteristics. These covariates were selected a priori because they represent caregiver-, family-, and educational-context characteristics that may be associated with opportunities and demands for family involvement. Age and number of children were included to account for differences in caregiver life stage and family caregiving demands, whereas educational level and school type were included because previous Chilean and Latin American research has highlighted the relevance of socioeconomic and institutional conditions for family participation in education (Gubbins et al., 2024; Sánchez Oñate et al., 2016; Gubbins & Otero, 2020; Santander Ramírez et al., 2022). The primary regression, mediation, and moderation models were estimated without these sociodemographic covariates; the complete covariate set was used in this adjusted regression model and again in the exploratory suppression sensitivity analysis.
(e) Moderation Analysis: To evaluate H2, caregiver gender was incorporated as a moderator of the ER–FIE relationship through the ER × Gender interaction term. Gender was coded as 1 = male and 2 = female. ER was mean-centered prior to constructing the interaction term to facilitate interpretation and reduce non-essential multicollinearity (Aiken & West, 1991). When a significant interaction was identified, simple-slope analyses were conducted separately for male and female caregivers. Although the ER × Gender interaction was estimated using the full sample (N = 219), the substantial imbalance between female (*n* = 181) and male (*n* = 38) caregivers was explicitly considered when interpreting the conditional slopes. Because the smaller male subgroup reduces the precision and potential stability of the male-specific estimate, this slope was treated as preliminary and hypothesis-generating. For the same reason, the complementary multigroup analysis was interpreted as exploratory rather than confirmatory. As a sensitivity analysis, the moderation model was additionally re-estimated after adjustment for age, number of children, educational level, and school type, using the same sociodemographic covariates included in the covariate-adjusted regression model. This analysis was conducted to examine whether the ER × Gender interaction remained statistically significant after accounting for these measured caregiver, family, and educational-context characteristics. Adjusted simple slopes were subsequently estimated separately for male and female caregivers.
(f) Multigroup Path Analysis: As a complementary robustness analysis, a multigroup path model was estimated by caregiver gender to examine whether the ER → DASS, DASS → FIE, and ER → FIE structural paths differed between male and female caregivers. An unconstrained model was compared with a model in which the structural regression paths were constrained to equality across groups. Follow-up score tests were used to identify the specific paths responsible for significant between-group differences.
(g) Alternative Directional Models: To examine whether an alternative directional specification provided a better representation of the observed associations, the proposed model (ER → DASS → FIE) was compared with a reverse model (ER → FIE → DASS). Models were compared using log-likelihood, Akaike Information Criterion (AIC), Bayesian Information Criterion (BIC), and sample-size-adjusted BIC (SABIC). Because both observed-variable models were saturated, conventional global fit indices were not interpreted as evidence favoring either directional specification.
(h) Exploratory suppression analysis: As a complementary exploratory analysis, regression models including ER alone and ER together with DASS were compared to examine a potential suppressor effect by evaluating changes in the ER regression coefficient and explained variance (*R*^2^) (MacKinnon et al., 2000). A sensitivity model additionally included age, number of children, educational level, and school type as covariates. Given its exploratory and non-hypothesized nature, this analysis was interpreted separately from the confirmatory hypothesis tests.

## 3. Results

### 3.1. Descriptive Statistics of Variables

Table 3 presents the descriptive statistics for all study variables (*n* = 219). Skewness and kurtosis analyses indicated that the variables did not show severe violations of normality assumptions (values between −2 and +2).

### 3.2. Main Association Between Emotion Regulation and Family Involvement in Education

Before testing the hypothesized mediation and moderation effects, the main effect of emotion regulation (ER) and family involvement in education (FIE) was examined using linear regression. The regression model was statistically significant, F(1, 217) = 15.30, *p* < 0.001, explaining 6.6% of the variance in FIE (*R*^2^ = 0.066; adjusted *R*^2^ = 0.062). Emotion regulation was positively and significantly associated with FIE (*B* = 0.252, *SE* = 0.064, β = 0.257, *t* = 3.92, *p* < 0.001, 95% CI [0.125, 0.378]), indicating that higher levels of emotion regulation were associated with greater family involvement in education.

### 3.3. Mediation Analysis: Testing the Proposed Indirect Pathway Through Psychological Distress

Following the examination of the main association between emotion regulation (ER) and family involvement in education (FIE), the proposed indirect pathway through psychological distress, operationalized as depressive, anxiety and stress symptomatology (DASS), was examined (H1). ER was significantly and negatively associated with DASS symptomatology (*B* = −0.654, *SE* = 0.095, *z* = −6.88, *p* < 0.001, 95% CI [−0.845, −0.473]), indicating that higher levels of emotion regulation were associated with lower psychological distress. However, when ER and DASS were simultaneously included as predictors of FIE, the DASS → FIE path was not statistically significant (*B* = −0.040, SE = 0.044, *z* = −0.91, *p* = 0.365, 95% CI [−0.124, 0.049]).

The direct effect of ER on FIE remained positive and statistically significant after accounting for DASS (*B* = 0.226, *SE* = 0.078, *z* = 2.91, *p* = 0.004, 95% CI [0.070, 0.376]). Importantly, the indirect effect of ER on FIE through DASS was not statistically significant (*B* = 0.026, *SE* = 0.029, *z* = 0.89, *p* = 0.374, 95% bootstrap CI [−0.032, 0.083]), as the confidence interval included zero. The total effect of ER on FIE was statistically significant (*B* = 0.252, *SE* = 0.077, *z* = 3.28, *p* = 0.001, 95% CI [0.100, 0.399]) (Table 4). Thus, although ER was significantly associated with lower psychological distress, there was no evidence that DASS statistically mediated the association between ER and FIE. Specifically, the DASS → FIE path and the indirect effect were both non-significant. Therefore, the findings provide evidence against the proposed indirect pathway in this sample, and H1 was not supported.

### 3.4. Model with Sociodemographic Covariates

Following the examination of the main and mediated relationships, a multiple linear regression model was estimated to assess whether the association between emotion regulation (ER) and family involvement in education (FIE) remained robust after accounting for sociodemographic characteristics. Age, number of children, educational level, and school type were included as covariates. The overall model was statistically significant, F(9, 209) = 2.72, *p* = 0.005, explaining 10.5% of the variance in FIE (*R*^2^ = 0.105; adjusted *R*^2^ = 0.066).

Importantly, ER remained positively and significantly associated with FIE after adjustment for the sociodemographic covariates (*B* = 0.270, *SE* = 0.067, β = 0.275, *t* = 4.03, *p* < 0.001, 95% CI [0.138, 0.402]). Neither age (*B* = −0.117, *p* = 0.170) nor number of children (*B* = 0.717, *p* = 0.363) was significantly associated with FIE. Likewise, none of the educational-level contrasts relative to incomplete secondary education reached statistical significance (all *p* ≥ 0.246).

Regarding school type, caregivers associated with private fee-paying schools reported significantly higher FIE scores than those associated with public schools (*B* = 3.162, *SE* = 1.570, *t* = 2.01, *p* = 0.045, 95% CI [0.067, 6.257]). The difference between subsidized private and public schools was not statistically significant (*B* = 2.499, *SE* = 1.496, *p* = 0.096, 95% CI [−0.450, 5.449]) (Table 5).

Overall, the positive ER-FIE association remained statistically significant after accounting for the sociodemographic characteristics included in the model, and its magnitude was similar to that observed in the unadjusted model (β = 0.257 vs. β = 0.275), indicating that covariate adjustment did not materially alter the main ER–FIE association.

### 3.5. Moderation Analysis: Role of Caregiver Gender

Finally, the moderating role of caregiver gender in the relationship between emotion regulation (ER) and family involvement in education (FIE) was examined (H2). Descriptively, male caregivers (*n* = 38) reported mean ER scores of *M* = 52.10 (*SD* = 7.84) and mean FIE scores of *M* = 65.10 (*SD* = 11.40), whereas female caregivers (*n* = 181) reported mean ER scores of M = 52.20 (*SD* = 9.13) and mean FIE scores of *M* = 70.30 (*SD* = 7.83). ER was mean-centered prior to constructing the interaction term, and male caregivers were initially specified as the reference group. The overall regression model including ER, caregiver gender, and the ER × Gender interaction was statistically significant, F(3, 215) = 15.60, *p* < 0.001, explaining 17.9% of the variance in FIE (*R*^2^ = 0.179; adjusted *R*^2^ = 0.167).

The ER × Gender interaction was statistically significant (*B* = 0.739, *SE* = 0.181, *t* = 4.08, *p* < 0.001, 95% CI [0.383, 1.096]), indicating that the association between ER and FIE differed significantly between male and female caregivers. Simple-slope analyses showed that, among male caregivers, ER was negatively and significantly associated with FIE (*B* = −0.394, *SE* = 0.169, *t* = −2.33, *p* = 0.021, 95% CI [−0.728, −0.061]). In contrast, among female caregivers, ER was positively and significantly associated with FIE (*B* = 0.345, *SE* = 0.065, *t* = 5.32, *p* < 0.001, 95% CI [0.217, 0.473]).

**Figure 1:**
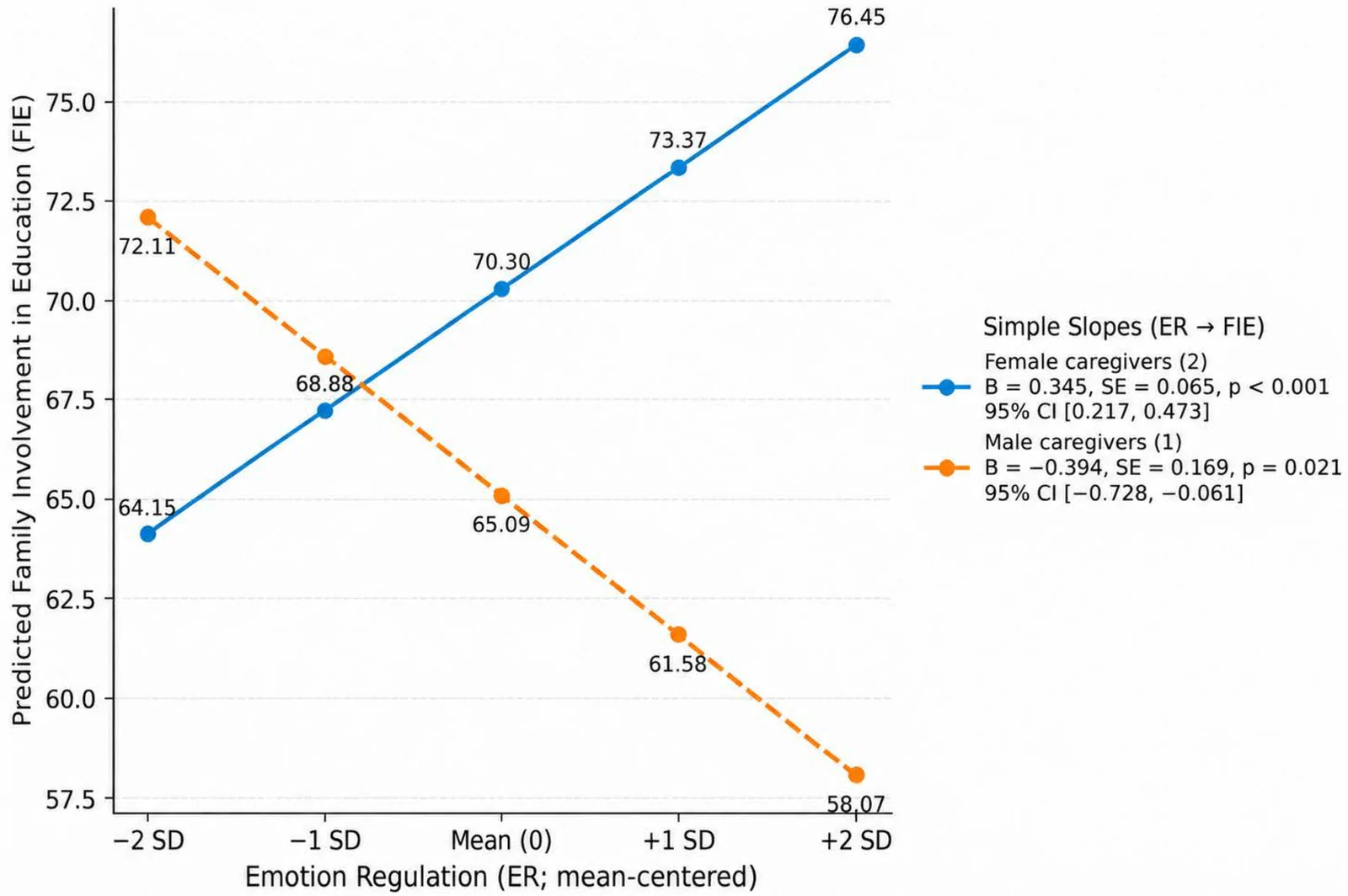
Simple Slopes of Emotional Regulation predicting Family Involvement in Education at each level of Gender. Note. ER = Emotion Regulation; FIE = Family Involvement in Education. ER was mean-centered prior to estimating the interaction. Gender was coded as 1 = male and 2 = female. Simple slopes represent the association between ER and FIE separately for male and female caregivers. Among female caregivers, ER was positively and significantly associated with FIE (*b* = 0.345, *SE* = 0.065, *p* < 0.001, 95% CI [0.217, 0.473]), whereas among male caregivers, ER was negatively and significantly associated with FIE (*b* = −0.394, *SE* = 0.169, *p* = 0.021, 95% CI [−0.728, −0.061]). The ER × Gender interaction was statistically significant (*b* = 0.739, *SE* = 0.181, *p* < 0.001, 95% CI [0.383, 1.096]). Predicted FIE values are displayed at the mean of ER and at ±1 and ±2 standard deviations from the mean.

Additionally, at the mean level of ER, female caregivers reported significantly higher FIE scores than male caregivers (*B* = 5.209, *SE* = 1.435, *t* = 3.63, *p* < 0.001, 95% CI [2.380, 8.037]). The complete moderation estimates are presented in Table 6, and the interaction pattern is illustrated in Figure 1. The statistically significant ER × Gender interaction was consistent with H2, indicating that the ER–FIE association differed according to caregiver gender in the present sample. However, because the male subgroup comprised only 38 caregivers, the group-specific slopes—particularly the negative slope observed among male caregivers—should be regarded as preliminary estimates requiring replication rather than as definitive evidence of opposite gender-specific mechanisms.

As a sensitivity analysis, the moderation model was re-estimated after adjustment for age, number of children, educational level, and school type. The adjusted model was statistically significant, F(11, 207) = 5.03, *p* < 0.001, explaining 21.1% of the variance in FIE (*R*^2^ = 0.211; adjusted *R*^2^ = 0.169). Importantly, the ER × Gender interaction remained statistically significant after adjustment (*B* = 0.695, *SE* = 0.184, t = 3.79, *p* < 0.001, 95% CI [0.333, 1.057]). Adjusted simple-slope analyses preserved the same directional pattern. Among male caregivers, ER remained negatively associated with FIE (*B* = −0.343, *SE* = 0.173, *t* = −1.99, *p* = 0.048, 95% CI [−0.684, −0.003]), whereas among female caregivers, ER remained positively associated with FIE (*B* = 0.352, *SE* = 0.067, *t* = 5.24, *p* < 0.001, 95% CI [0.219, 0.484]). Given the small male subgroup and the borderline statistical significance of the adjusted male-specific slope, this estimate should continue to be interpreted cautiously.

As a complementary robustness analysis, a multigroup path analysis was conducted to examine whether the structural relationships differed across caregiver gender. Constraining the three structural paths to equality across male and female caregivers resulted in a significant overall test of cross-group equality, *χ*^2^(3) = 8.80, *p* = 0.032. Follow-up score tests indicated that the cross-group difference was specifically attributable to the ER → FIE path, *χ*^2^(1) = 7.90, *p* = 0.005. In contrast, no significant gender differences were observed for the ER → DASS path, *χ*^2^(1) = 0.002, *p* = 0.963, or the DASS → FIE path, *χ*^2^(1) = 1.29, *p* = 0.256. Although the ER → FIE path differed statistically across groups in this exploratory analysis, the small male subgroup limits the precision and stability of the multigroup estimates. Consequently, these results should not be considered confirmatory evidence of structural differences between male and female caregivers.

### 3.6. Summary of Mediation and Moderation Findings

**Figure 2:**
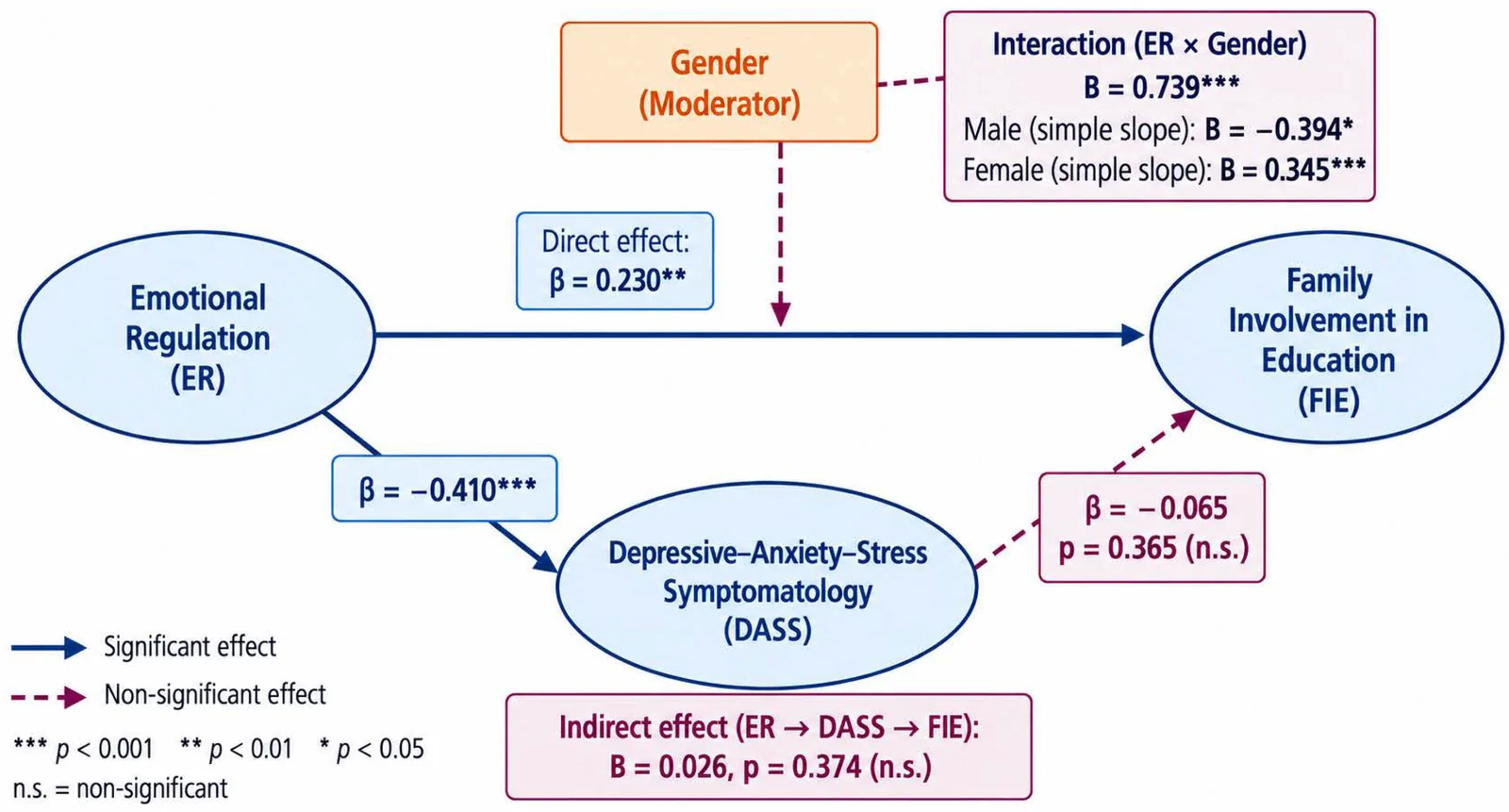
Summary Diagram of the Mediation and Moderation Findings. Note. β values represent standardized coefficients and B values represent unstandardized coefficients. Solid arrows indicate statistically significant associations, whereas dashed arrows indicate non-significant associations. The figure summarizes results obtained from separately estimated mediation and moderation analyses; it does not represent a single integrated moderated mediation model.

The mediation and moderation analyses were estimated separately rather than as a single integrated moderated mediation model. This section therefore summarizes their findings jointly to provide an overall representation of the hypothesized relationships. The mediation analysis showed that ER was negatively associated with DASS (β = −0.410, *p* < 0.001), whereas DASS was not significantly associated with FIE (β = −0.065, *p* = 0.365). ER maintained a positive and significant direct association with FIE (β = 0.230, *p* = 0.004). Consequently, the indirect effect of ER on FIE through DASS was not statistically significant (*B* = 0.026, *p* = 0.374), providing no evidence for the proposed mediation mechanism. In contrast, caregiver gender significantly moderated the association between ER and FIE (*B* = 0.739, *SE* = 0.181, *p* < 0.001). As a complementary analysis, the multigroup analysis showed that the structural paths were not fully equivalent across gender groups, *χ*^2^(3) = 8.80, *p* = 0.032. Specifically, the ER → FIE path differed significantly between male and female caregivers, *χ*^2^(1) = 7.90, *p* = 0.005, whereas the ER → DASS, χ^2^(1) = 0.002, *p* = 0.963, and DASS → FIE paths, *χ*^2^(1) = 1.29, *p* = 0.256, did not differ significantly between groups. Taken together, the separately estimated analyses provided no evidence for the proposed indirect pathway through DASS, while supporting a gender-dependent association between ER and FIE. No moderated mediation model was formally tested in the present study (Figure 2).

### 3.7. Comparison of Alternative Directional Models

To examine whether an alternative directional specification provided a better representation of the observed associations, the proposed mediation model (ER → DASS → FIE) was compared with a reverse model (ER → FIE → DASS). Both models were saturated and yielded identical log-likelihood and information criteria (AIC = 4891.46, BIC = 4921.96, SABIC = 4893.44) (Table 7). Consequently, global fit indices cannot be used to distinguish between these alternative directional specifications.

In the reverse model, ER was significantly associated with FIE (β = 0.257, *p* < 0.001) and remained negatively associated with DASS after accounting for FIE (β = −0.395, *p* < 0.001). However, FIE was not significantly associated with DASS (β = −0.058, *p* = 0.391). Thus, the reverse specification did not provide evidence for an alternative indirect pathway. Given the cross-sectional nature of the data and the statistical equivalence of the saturated models, these analyses should not be interpreted as evidence of causal direction. Rather, they indicate that neither directional specification supports an indirect association involving DASS and FIE.

### 3.8. Exploratory Suppression Analysis

An exploratory regression analysis was conducted to examine whether DASS symptomatology exerted a suppressor effect on the association between emotion regulation (ER) and family involvement in education (FIE). In the model including ER alone, ER was significantly associated with FIE (*B* = 0.252, *R*^2^ = 0.066). After adding DASS, the ER coefficient decreased slightly to *B* = 0.226, while DASS showed a small non-significant association with FIE (*B* = −0.040, *p* = 0.364), and the explained variance increased only marginally (*R*^2^ = 0.070; Δ*R*^2^ = 0.004). This pattern provides no meaningful evidence of a suppressor effect.

As a sensitivity analysis, age, number of children, educational level, and school type were additionally included as covariates. ER remained positively and significantly associated with FIE (*B* = 0.247, *p* < 0.001), whereas DASS remained non-significant (*B* = −0.038, *p* = 0.400). The fully adjusted model explained 10.8% of the variance in FIE (*R*^2^ = 0.108). Overall, these findings indicate that the ER–FIE association is robust to the inclusion of DASS and the selected sociodemographic covariates and provide no evidence that DASS acts as a suppressor variable (Table 8). The inclusion of the same sociodemographic covariates in the sensitivity model did not materially alter the pattern of results: ER remained significantly associated with FIE, whereas DASS remained non-significant.

Taken together, the results indicate that emotion regulation (ER) is positively associated with family involvement in education (FIE) overall. No evidence was found that depressive, anxiety and stress symptomatology (DASS) mediates or suppresses this association, and the alternative directional model did not support an indirect pathway involving FIE and DASS. In contrast, caregiver gender significantly moderated the ER–FIE relationship, with ER being positively associated with FIE among female caregivers and negatively associated with FIE among male caregivers. Overall, these findings support a gender-moderated direct association between ER and FIE rather than the hypothesized mediation mechanism (Figure 2).

## 4. Discussion

The present study examined the association between parental emotion regulation (ER) and family involvement in education (FIE), specifically evaluating the hypothesized mediating role of depressive, anxiety and stress symptomatology (DASS) and the moderating effect of gender. The results partially support the proposed hypotheses, revealing a complex pattern of relationships that provides preliminary evidence for the field of educational and family psychology.

First, a significant and positive direct association was found between parental emotion regulation and family involvement after accounting for DASS (β = 0.230, *p* = 0.004). This finding is consistent with previous literature indicating that emotion regulation skills may facilitate persistence in challenging tasks, parental stress management, and the maintenance of collaborative interactions with the school (Bridgett et al., 2015). Parents who report greater ability to identify, understand, and regulate their emotions may have psychological resources that allow them to engage more consistently and effectively in their children’s education, potentially overcoming barriers such as fatigue, frustration, or communication conflicts (Meyer et al., 2014). These findings are consistent with recent research highlighting emotion regulation as a relevant psychological correlate of family involvement in contexts of educational vulnerability (Lara & Saracostti, 2019; De Raeymaecker & Dhar, 2022; Zulauf-McCurdy et al., 2024).

From a Chilean perspective, this association is particularly relevant because family involvement develops within educational contexts in which opportunities for participation may vary according to socioeconomic and institutional conditions. Previous Chilean research has shown that parental expectations, perceived self-efficacy, access to school-provided information, and socioeconomic circumstances can shape both home- and school-based forms of involvement (Sánchez Oñate et al., 2016; Gubbins & Otero, 2020; Santander Ramírez et al., 2022). Therefore, the positive ER–FIE association observed in the present study should not be interpreted as suggesting that family involvement depends exclusively on individual caregiver characteristics. Rather, parental emotional resources may operate within a broader relational and institutional context that can either facilitate or constrain their translation into educational involvement.

Contrary to H1, the proposed indirect pathway through psychological distress was not supported. ER was significantly and negatively associated with DASS (β = −0.410, *p* < 0.001), indicating that higher emotion regulation was associated with lower psychological distress. However, when ER and DASS were simultaneously included as predictors of FIE, the DASS → FIE path was not statistically significant (β = −0.065, *p* = 0.365). Consistent with this result, the indirect effect of ER on FIE through DASS was also not statistically significant (*B* = 0.026, *p* = 0.374), and its bootstrap confidence interval included zero. Thus, the significant ER → DASS association should be distinguished from evidence of mediation: although ER was associated with psychological distress, there was no evidence that DASS statistically transmitted the association between ER and FIE in the present sample. These findings therefore provide evidence against the hypothesized indirect pathway in this sample rather than evidence that DASS functions as a meaningful mediator. This null indirect effect should be considered an informative finding rather than evidence that psychological distress is generally unrelated to family involvement.

One possible alternative interpretation not tested in this study is that the association between symptomatology and involvement could vary according to other factors, such as perceived social support, parental self-efficacy, or child characteristics (Silk et al., 2011; Goodman et al., 2011). Although it was not possible to test this explanation with our data, it is also plausible that the association between mental health and family involvement may be nonlinear or threshold-based, manifesting only when symptomatology reaches clinical levels, which was not assessed in this study. Recent studies have observed that the relationship between internalizing symptoms and educational involvement is moderated by the perception of parental self-efficacy (García Linares et al., 2011; Peris et al., 2018), which could explain the absence of direct mediation in our model; however, this possibility requires empirical testing in future research.

A statistically significant ER × Gender interaction was observed, consistent with H2, indicating that the ER–FIE association differed between male and female caregivers in the present sample (*B* = 0.739, *SE* = 0.181, *p* < 0.001, 95% CI [0.383, 1.096]). Simple-slopes analysis showed estimated associations that differed in direction across caregiver gender: among male caregivers, ER was negatively and significantly associated with FIE (*B* = −0.394, *SE* = 0.169, *p* = 0.021, 95% CI [−0.728, −0.061]), whereas among female caregivers, ER was positively and significantly associated with FIE (*B* = 0.345, *SE* = 0.065, *p* < 0.001, 95% CI [0.217, 0.473]). However, given the relatively small male subsample (*n* = 38), the male-specific estimate and the apparent difference in direction between groups should be regarded as preliminary and interpreted cautiously. In particular, the negative slope observed among male caregivers should not be interpreted as evidence that greater emotion regulation reduces family involvement.

One possible theoretical interpretation of the observed pattern is consistent with perspectives suggesting that emotional experiences and parental roles may be shaped differently according to gender (Chaplin & Aldao, 2013). Consistent with Tamres et al. (2002), women may more frequently rely on emotion regulation strategies centered on expression and support-seeking, which could translate more directly into educational involvement behaviors, whereas men may rely relatively more on internalized or instrumental problem-solving strategies. These explanations were not directly tested in the present study and should therefore be regarded as theoretical possibilities rather than demonstrated mechanisms underlying the interaction.

As an exploratory sensitivity analysis, a complementary multigroup analysis was conducted. The ER → FIE path differed significantly between male and female caregivers, *χ*^2^(1) = 7.90, *p* = 0.005, whereas the ER → DASS and DASS → FIE paths did not significantly differ between groups. Given the small male subgroup, these multigroup findings should not be considered independent confirmatory evidence of gender-specific structural differences, but rather complementary evidence requiring replication.

Overall, the findings suggest that caregiver gender may be a relevant factor to consider in future research examining the relationship between emotion regulation and family educational involvement. Recent research in Latin American contexts has also reported gender-related differences in emotion regulation and parental involvement (Orgilés et al., 2023; Oyarzún-Farías et al., 2024). However, the present findings do not provide sufficient evidence to recommend gender-differentiated psychoeducational interventions. Replication with larger and more gender-balanced samples is necessary before drawing substantive conclusions regarding gender-specific patterns or translating these findings into differentiated intervention strategies.

Regarding the control variables, age, number of children, and educational-level contrasts were not significantly associated with FIE. For school type, however, caregivers of students attending private fee-paying schools reported significantly higher FIE scores than caregivers associated with public schools (*B* = 3.162, *p* = 0.045), whereas the difference between subsidized private and public schools was not statistically significant. Thus, the covariate-adjusted analysis generally supported the robustness of the ER–FIE association, while also identifying a specific association between private fee-paying school attendance and family involvement.

This school-type difference is particularly relevant when interpreted within the Chilean educational context. However, the higher FIE scores observed among caregivers of students attending private fee-paying schools should not be interpreted as indicating that families associated with public schools place less value on their children’s education. Previous Chilean research suggests that family educational involvement is socially and institutionally patterned: socioeconomic constraints, time availability, parental expectations and self-efficacy, access to information, and the opportunities for participation created by schools can influence both how and where caregivers become involved in their children’s education (Sánchez Oñate et al., 2016; Gubbins & Otero, 2020; Santander Ramírez et al., 2022). Accordingly, differences in visible participation across school types may partly reflect unequal opportunity structures and different family–school interaction practices rather than intrinsic differences in parental commitment.

This interpretation should nevertheless remain cautious. School type cannot be considered a direct proxy for household socioeconomic status, which was not directly measured in the present study, and the observed private–public difference was relatively modest and should be replicated in larger and more representative samples. Therefore, the present findings cannot determine whether the school-type association reflects socioeconomic resources, institutional practices, family characteristics, or a combination of these factors.

School type and educational level were included as covariates rather than moderators in the present analytical strategy. Although multicategorical variables can be examined as moderators through appropriate coding and interaction terms, such analyses were beyond the scope of the present hypotheses and would require adequate subgroup sizes to obtain stable conditional estimates. Consequently, the present findings should not be interpreted as demonstrating that the ER–FIE association is invariant across educational or socioeconomic contexts. Nevertheless, the fact that ER remained positively associated with FIE after adjustment for the sociodemographic characteristics included in the model suggests that parental emotional resources may be relevant even when some contextual differences are statistically accounted for. This interpretation is consistent with research suggesting that parental psychological resources may represent relevant cross-cutting correlates of educational involvement, including in contexts of educational vulnerability (Formoso-Silva et al., 2025; Fuentes-Vilugrón et al., 2025).

More broadly, these findings reinforce the need for a relational interpretation of family involvement in Chile and other Latin American contexts. Family involvement should not be conceptualized solely as a behavior that individual caregivers either choose or fail to perform; it is also shaped by the opportunities, expectations, communication practices, and forms of recognition established by educational institutions. Consequently, initiatives aimed at strengthening family–school relationships may need to combine support for caregivers’ emotional and psychological resources with institutional strategies that reduce participation barriers, diversify opportunities for involvement, and recognize educational support practices occurring both inside and outside the school. This perspective is consistent with the growing Latin American literature emphasizing the contextual and multidimensional nature of family participation in education (Gubbins et al., 2024).

The exploratory suppression analysis revealed only minimal changes when including DASS in the regression model: the direct ER-FIE coefficient decreased slightly (from B = 0.252 to B = 0.226), and the explained variance increased marginally (Δ*R*^2^ = 0.004). Given the minimal magnitude of these changes, the observed pattern does not provide meaningful evidence of suppression. Therefore, this exploratory analysis does not support the presence of a suppressor effect in our sample (see Table 8). Taken together, the findings suggest that parental emotion regulation represents a relevant psychological correlate of family educational involvement, but its role should be interpreted within the social and institutional conditions in which family–school relationships occur. In Chile, and potentially in other Latin American educational systems characterized by socioeconomic differentiation, strengthening family involvement may therefore require attention to both caregivers’ psychological resources and the conditions created by schools for meaningful and equitable participation.

### 4.1. Theoretical and Practical Implications

From a practical perspective, the findings suggest that parental emotion regulation may represent a relevant psychological correlate to consider when examining family involvement in education, rather than treating family participation exclusively as a matter of parental willingness or motivation. However, because the present study is cross-sectional and correlational and did not manipulate emotion regulation, the findings do not establish that improving parental emotion regulation would increase family involvement or reduce psychological distress. Accordingly, parental emotion regulation should not yet be considered an empirically established intervention target, and the present results do not provide a sufficient basis for recommending specific emotion-regulation interventions or gender-differentiated strategies. Rather, these findings support the need for longitudinal and intervention studies to determine whether changes in ER precede or contribute to changes in family involvement and psychological distress. The observed gender-specific pattern should likewise be replicated in larger and more gender-balanced samples before any substantive or applied conclusions are drawn.

Finally, the evaluation of family involvement in education should consider not only observable behaviors but also the parental psychological resources that facilitate or limit them, as well as the institutional opportunities and constraints that shape how such involvement is expressed. This broader perspective is particularly relevant in educational systems characterized by socioeconomic differentiation, such as Chile, because lower levels of visible school-based participation should not automatically be interpreted as lower parental commitment to children’s education.

### 4.2. Limitations

This study has several limitations that should be considered when interpreting the results: a) the cross-sectional design does not allow for inferring causality or directionality in the relationships. Future research should implement longitudinal or experimental designs to establish temporal sequences and causal effects; b) the data depend on the subjective perception of participants, potentially being subject to social desirability or memory biases. In addition, ER, psychological distress, and FIE were assessed through self-report measures completed by the same caregivers at the same time point. Consequently, shared-source measurement and common method variance cannot be ruled out and may have contributed to the observed covariation among these constructs, potentially affecting the magnitude of the estimated associations. Future studies should incorporate multiple informants, such as other caregivers, children, or educational professionals, as well as behavioral or school-based indicators of family involvement and longitudinal measurement designs to reduce reliance on a single source and time point; c) generalization of the findings is limited by the non-probabilistic sampling and sample size (*n* = 219), which may limit the detection and precise estimation of small effects and restrict the generalizability of the findings to other populations and sociocultural contexts; and d) the marked gender imbalance in the sample (82.6% female, 17.4% male, with only *n* = 38 male caregivers) reduces the precision and potential stability of the male-specific estimates derived from the moderation analysis. Although regression models with interaction terms can accommodate unequal group sizes, the relatively small male subsample increases uncertainty surrounding the male-specific slope and limits confidence in the replicability of the observed gender-dependent pattern. This consideration is particularly important because the estimated ER–FIE associations differed in direction across male and female caregivers. The small male subsample also places additional constraints on the interpretation of the complementary multigroup analysis (Kline, 2023). Although this exploratory analysis yielded a statistically significant difference in the ER–FIE path between groups, it should not be regarded as independent confirmatory evidence of gender-specific structural differences. Consequently, the moderation findings should be considered preliminary and hypothesis-generating rather than definitive evidence of opposite gender-specific mechanisms. Future research should employ larger and more gender-balanced samples to determine whether the observed ER × Gender interaction and the corresponding group-specific slopes are replicable.

## 5. Conclusions

This study provides evidence of a positive overall association between parental emotion regulation and family involvement in education. This association remained statistically significant after accounting for DASS symptomatology and sociodemographic characteristics. DASS symptomatology did not statistically mediate the ER–FIE association, indicating that the hypothesized indirect pathway through psychological distress was not supported in the present sample. A statistically significant ER × Gender interaction was also observed, with estimated associations differing in direction across caregiver gender: positive among female caregivers and negative among male caregivers. However, given the relatively small male subsample, these gender-specific estimates should be regarded as preliminary and interpreted cautiously. The complementary multigroup analysis showed a similar between-group difference in the ER–FIE path, but, given the limited number of male caregivers, it should be considered exploratory rather than independent confirmatory evidence.

The absence of a significant indirect effect through DASS suggests that the association between parental emotion regulation and family involvement was not statistically accounted for by depressive, anxiety and stress symptomatology in this sample. Other potential pathways, such as parental self-efficacy, perceived social support, or specific parenting practices, may warrant examination in future research.

From a theoretical perspective, these findings contribute to the integration of Gross’s process model of emotion regulation with conservation of resources theory, showing that parental emotion regulation was associated with both educational involvement and psychological distress, while providing no evidence that DASS symptomatology constituted the mediating mechanism proposed in the present study. The observed interaction with caregiver gender additionally suggests that gender-related differences may warrant consideration in future models of family involvement, although the present data are insufficient to establish distinct gender-specific mechanisms. Importantly, the findings also support a contextually situated interpretation of family educational involvement, in which caregivers’ psychological resources operate within broader socioeconomic and institutional environments that may facilitate or constrain participation.

From a practical standpoint, the overall positive association between emotion regulation and family involvement suggests that parental emotional resources may be relevant when examining family–school collaboration. However, the present cross-sectional findings do not provide a basis for recommending gender-differentiated interventions. The observed gender-specific pattern requires replication in larger and more gender-balanced samples before substantive conclusions regarding differential patterns among male and female caregivers can be drawn. Furthermore, although DASS did not mediate the ER–FIE association, ER was significantly associated with lower DASS symptomatology. This finding supports further investigation of emotion regulation in relation to both parental psychological well-being and educational involvement, particularly through longitudinal or intervention studies capable of testing temporal and causal relationships.

In the Chilean context, these findings should be interpreted alongside the social and institutional conditions under which family involvement occurs. The higher FIE observed among caregivers associated with private fee-paying schools should not be interpreted as evidence of lower educational commitment among families linked to public schools, but rather as a finding that may reflect differences in socioeconomic resources, institutional practices, access to information, and opportunities for participation. More broadly, in Chile and other Latin American contexts, strengthening family–school relationships may therefore require attention to both caregivers’ emotional and psychological resources and the institutional conditions that enable meaningful and equitable participation.

Despite these contributions, the results should be interpreted in light of several limitations, including the cross-sectional design, non-probabilistic sampling, and marked gender imbalance in the sample, which preclude causal inferences and limit generalizability. Future research should employ longitudinal or experimental designs, incorporate larger and more gender-balanced samples, and explore potential moderators and mediators not examined in this study, such as parental self-efficacy, perceived social support, and cultural factors. In conclusion, this study contributes an integrated analytical examination of the associations among parental emotion regulation, psychological distress, and family involvement in education, highlighting parental emotion regulation as a relevant correlate of both educational involvement and psychological symptomatology. The findings also identify a preliminary gender-dependent pattern in the ER–FIE association that requires independent replication before firm conclusions regarding caregiver-gender differences can be established. Overall, the results suggest that family involvement in education should not be understood exclusively as an individual caregiver characteristic, but as a phenomenon emerging from the interaction between parental psychological resources and the social and institutional conditions in which family–school relationships develop. This perspective may be particularly relevant for Chile and other Latin American educational systems characterized by substantial socioeconomic differentiation, and provides a basis for future research aimed at identifying both individual and contextual conditions that promote equitable family participation in education.

## Figures and Tables

**Table 1:** Sociodemographic Characteristics of the Sample.

Variable	Category	*n*	%
Gender	Male	38	17.4
	Female	181	82.6
Age	*M (SD)*	38.60	(7.39)
	Range	21–68	
Number of children	*M (SD)*	1.71	(0.77)
	Range	1–5	
Educational Level	Incomplete secondary education	2	0.9
	Complete secondary education	20	9.1
	Technical higher education	31	14.2
	University education	106	48.4
	Postgraduate education	60	27.4
School Type	Public	59	26.9
	Subsidized	85	38.8
	Private	75	34.2

Note. M = Mean; SD = Standard Deviation. No participants reported incomplete or complete primary education.

**Table 2:** Internal Consistency of the Study Scales.

Scale	Dimension	α	ω
DASS-21	Depression	0.907	0.908
	Anxiety	0.889	0.894
	Stress	0.893	0.895
	Total	0.914	0.915
RE-MESACTS	Positive Emotion Expression	0.877	0.880
	Self-Regulation of Negative Emotions	0.784	0.787
	Self-Regulation Strategies	0.841	0.846
	Emotion Identification	0.738	0.742
	Total	0.792	0.794
EPFE	Home Support	0.798	0.826
	Democratic Participation	0.818	0.825
	School-Family Communication	0.810	0.835
	Total	0.798	0.826

Note. DASS-21 = Depression, Anxiety and Stress Scale-21; RE-MESACTS = Emotion Regulation Scale for Adults (Spanish version); EPFE = Family Involvement in Education Scale. α = Cronbach’s alpha; ω = McDonald’s omega. All coefficients are based on the present study sample (*n* = 219).

**Table 3:** Descriptive Statistics of Study Variables.

Variable	*n*	*M*	*SD*	Min	Max	Skewness	Kurtosis
EP (ER)	219	13.59	2.65	4	16	−1.03	0.32
AN (ER)	219	14.81	2.87	7	20	−0.10	−0.42
EA (ER)	219	11.69	2.80	5	16	−0.20	−0.82
IE (ER)	219	12.10	3.03	4	16	−0.34	−0.72
D (DASS)	219	4.80	4.68	0	18	0.86	−0.34
A (DASS)	219	5.71	5.44	0	21	0.92	−0.03
E (DASS)	219	9.13	5.36	0	21	0.25	−0.91
AE (FIE)	219	35.79	4.01	12	40	−1.77	5.31
PE (FIE)	219	19.28	3.99	6	24	−0.84	0.13
CE (FIE)	219	14.36	2.36	4	16	−1.87	3.74
Age	219	38.60	7.39	21	68	0.85	1.15

Note. ER = total score of the RE-MESACT scale; EP = positive emotion expression; AN = negative emotion self-regulation; EA = self-regulation strategies; IE = emotion identification; DASS = total score of the DASS-21 scale; D = depression; A = anxiety; E = stress; AE = home support; PE = democratic participation; CE = school-family communication; FIE = total score of the Family Involvement in Education Scale (EPFE).

**Table 4:** Mediation Analysis of the Proposed Indirect Pathway through DASS.

Effect/Path	*B*	*SE*	*z*	*p*	95% CI
Path estimates					
ER → DASS	−0.654	0.095	−6.88	<0.001	[−0.845, −0.473]
DASS → FIE	−0.040	0.044	−0.91	0.365	[−0.124, 0.049]
ER → FIE (direct)	0.226	0.078	2.91	0.004	[0.070, 0.376]
Effects					
Indirect	0.026	0.029	0.89	0.374	[−0.032, 0.083]
Direct	0.226	0.078	2.91	0.004	[0.070, 0.376]
Total	0.252	0.077	3.28	0.001	[0.100, 0.399]

Note. ER = Emotion Regulation; DASS = Depressive, Anxiety, and Stress Symptomatology; FIE = Family Involvement in Education. Confidence intervals for the indirect effect were estimated using 5000 bootstrap resamples.

**Table 5:** Multiple Linear Regression Model Including Sociodemographic Covariates.

Predictor	*B*	*SE*	*t*	*p*	95% CI
ER	0.270	0.067	4.03	<0.001	[0.138, 0.402]
Age	−0.117	0.085	−1.38	0.170	[−0.285, 0.051]
Number of children	0.717	0.786	0.91	0.363	[−0.832, 2.266]
Complete secondary education ^1^	−7.548	6.494	−1.16	0.246	[−20.350, 5.255]
Technical higher education ^1^	−6.846	6.351	−1.08	0.282	[−19.365, 5.674]
University education ^1^	−6.390	6.267	−1.02	0.309	[−18.745, 5.964]
Postgraduate education ^1^	−6.199	6.282	−0.99	0.325	[−18.584, 6.185]
Subsidized private school ^2^	2.499	1.496	1.67	0.096	[−0.450, 5.449]
Private fee-paying school ^2^	3.162	1.570	2.01	0.045	[0.067, 6.257]

Note: ER = Emotion Regulation; ^1^ Reference category for educational level = incomplete secondary education. Categories corresponding to incomplete and complete primary education were not represented in the analyzed sample. ^2^ Reference category for school type = public school. *R*^2^ = 0.105; adjusted *R*^2^ = 0.066; F(9, 209) = 2.72, *p* = 0.005.

**Table 6:** Moderating Effect of Caregiver Gender on the ER–FIE Relationship.

Predictor/Effect	*B*	*SE*	*t*	*p*	95% CI
Panel A. Primary unadjusted moderation model					
ER among male caregivers	−0.394	0.169	−2.33	0.021	[−0.728, −0.061]
ER among female caregivers	0.345	0.065	5.32	<0.001	[0.217, 0.473]
Gender (Female − Male) ^1^	5.209	1.435	3.63	<0.001	[2.380, 8.037]
ER × Gender	0.739	0.181	4.08	<0.001	[0.383, 1.096]
Panel B. Covariate-adjusted sensitivity analysis					
ER among male caregivers	−0.343	0.173	−1.99	0.048	[−0.684, −0.003]
ER among female caregivers	0.352	0.067	5.24	<0.001	[0.219, 0.484]
ER × Gender	0.695	0.184	3.79	<0.001	[0.333, 1.057]

Note. ER = Emotion Regulation. ER was mean-centered prior to constructing the interaction term. Male caregivers were used as the reference group in the primary moderation model. The female simple slope in Panel A was obtained by reparameterizing the same model with female caregivers as the reference group. ^1^ The gender coefficient represents the female–male difference in FIE at the mean level of ER. Panel B presents the sensitivity analysis adjusted for age, number of children, educational level, and school type. Adjusted simple slopes were estimated separately for male and female caregivers. For the primary unadjusted model, *R*^2^ = 0.179, adjusted *R*^2^ = 0.167, F(3, 215) = 15.60, *p* < 0.001. For the covariate-adjusted model, *R*^2^ = 0.211, adjusted *R*^2^ = 0.169, F(11, 207) = 5.03, *p* < 0.001.

**Table 7:** Fit Comparison Between Alternative Directional Models.

Model	Log-Likelihood	AIC	*BIC*	*SABIC*
Proposed: ER → DASS → FIE	−2436.73	4891.46	4921.96	4893.44
Reverse: ER → FIE → DASS	−2436.73	4891.46	4921.96	4893.44

Note. ER = Emotion Regulation; DASS = Depressive, Anxiety and Stress Symptomatology; FIE = Family Involvement in Education; AIC = Akaike Information Criterion; BIC = Bayesian Information Criterion; SABIC = sample-size adjusted Bayesian Information Criterion. Both models were saturated (df = 0); therefore, conventional global fit indices cannot discriminate between them.

**Table 8:** Exploratory Regression Analysis to Evaluate Possible Suppressor Effect of DASS.

Model	B (ER)	B (DASS)	*R* ^2^	Δ*R*^2^
ER only	0.252	–	0.066	–
ER + DASS	0.226	−0.040	0.070	0.004
ER + DASS + Covariates	0.247	−0.038	0.108	0.038

Note. ER = Emotion Regulation; DASS = Depressive, Anxiety and Stress Symptomatology. Covariates = age, number of children, educational level, and school type. ΔR^2^ represents the increase in explained variance relative to the preceding model.

## Data Availability

The main data supporting the results of this study are included within the article.
